# Development and validation of novel automatable assay for cholesterol efflux capacity

**DOI:** 10.1042/BSR20221519

**Published:** 2023-02-07

**Authors:** Yume Mutsuda, Tsunehiro Miyakoshi, Yuna Horiuchi, Takahiro Kameda, Minoru Tozuka, Ryunosuke Ohkawa

**Affiliations:** 1 Department of Analytical Laboratory Chemistry, Graduate School of Medical and Dental Sciences, Tokyo Medical and Dental University (TMDU), Tokyo, Japan; 2 Division of Clinical Laboratory, Juntendo University Urayasu Hospital, Chiba, Japan; 3 Department of Clinical Laboratory Technology, Juntendo University, Chiba, Japan; 4 Life Science Research Center, Nagano Children’s Hospital, Nagano, Japan

**Keywords:** automation, cholesterol efflux capacity, high-density lipoprotein

## Abstract

During the past decade, evaluation of high-density lipoprotein (HDL) functionality has been well studied for predicting cardiovascular disease (CVD) risk. Cholesterol efflux capacity (CEC) is the strongest candidate as the biomarker out of various HDL antiatherosclerotic functions. However, CEC has not yet been introduced clinically because of several technical issues, including the use of radioactive materials and differentiated cells in the assay. Previously, our laboratory developed a radioisotope- and cell-free CEC assay called the immobilized liposome-bound gel beads (ILGs) method to replace the conventional method. However, the separation process of the supernatant was not suitable for installation in an automatic analyzer. The present study aims to develop a new method that is easier to operate. We assumed that the use of magnetic beads instead of gel beads would enable the skip of the centrifugal process. First, similar to the ILG method, porous magnetic beads were treated with liposomes containing fluorescently labeled cholesterol. Fluorescence was observed inside the magnetic beads, and almost the same amount of liposomes as in the ILG method was immobilized successfully. These immobilized liposome-bound magnetic beads (ILMs) were available for CEC assay when HDL and apolipoprotein B-100-depleted serum (BDS) were used as cholesterol acceptors. The ILM method showed sufficient basic performance and a good correlation with the ILG method. Furthermore, when the CEC of 15 serum samples from healthy subjects was measured, a good correlation between HDL-cholesterol level and the ILG method was confirmed. Thus, it was confirmed that the ILM method was successfully developed and could be automated.

## Introduction

In general, it is widely known that high-density lipoprotein (HDL) has multiple antiatherosclerotic functions such as cholesterol efflux capacity (CEC) [1], antioxidant- [2], anti-inflammatory- [3], and antithrombotic- [4] activities, and so on. Therefore, the diagnostic criteria for dyslipidemia include the value of HDL cholesterol (HDL-C) concentration, which reflects the amount of HDL [5]. In addition, several clinical trials of HDL-C-increasing drugs, such as niacin and cholesteryl ester transfer protein inhibitors, have been attempted [6]. However, it has also been reported that increased HDL-C does not necessarily reduce the risk of cardiovascular disease (CVD) [7]. HDL is a collective term for heterogeneous particles with various protein and lipid compositions, and each HDL has a different antiatherosclerosis function. Hence, HDL qualifications should be performed to predict the risk of CVD in addition to quantification.

CEC is one of the most important antiatherosclerotic HDL functions. Atherosclerosis, the dominant cause of CVD, is induced by cholesterol accumulation in foam cells. HDL-CEC is a capacity that extracts the loaded cholesterol from foam cells, resulting in the reduction in atherosclerotic lesions. With regard to an assessment for the HDL-CEC, several specific pathways in cholesterol efflux from cholesterol-enriched macrophages are involved [8]. One of the pathways is ATP-binding cassette transporter (ABC) A1 pathway, which is an important transporter in HDL biogenesis: lipid-poor apolipoprotein A-I (apoA-I) acquires free cholesterol from hepatic and peripheral tissue via ABCA1 [9]. The other pathway is also active pathway via ABCG1 where mature HDL promotes the cholesterol efflux, which does not require direct binding of HDL to the ABCG1 [10]. The last two pathways are passive processes called scavenger receptor class B type 1 (SR-B1) pathway and aqueous diffusion pathway. Both pathways mediate bidirectional flux between macrophages and mature HDL [10].

A previous study demonstrated that CEC quantifying total efflux by above pathways (34% by ABCA1, 20% by SR-B1, and 46% by ABCG1, aqueous diffusion, or undiscovered pathways [11]) was a strong inverse predictor of CVD status [12]. In addition, another study evaluated ABCA1-mediated CEC reported that the high CEC group had a 67% lower CVD risk than the low CEC group, independent of the HDL-C level [13]. Consequently, CEC is one of the functions that has received particular attention in recent years and is considered to be one of the new biomarkers for atherosclerosis.

However, since CEC assays are performed using differentiated cells and radioisotope-labeled cholesterol [14], there are several issues such as time consumption, complicated handling, and safety to be introduced into a clinical setting. To solve this problem, we developed a novel radioisotope- and cell-free CEC assay, the immobilized liposome-bound gel beads (ILGs) method [15], to replace the conventional method, and demonstrated that the ILG method showed a good correlation with the conventional cell-based method [15]. Moreover, this method was extremely superior to the conventional method in aspects of repeatability and shorten turnaround time [16]. Thus, the ILG method enables safer, simpler, and more accurate measurements than the conventional method to evaluate CEC of serum from large numbers of subjects for running health screening or making diagnosis to predict the CVD risk. However, the separation process of the supernatant using a spin-down centrifuge was not suitable for installation on an automatic analyzer. In the present study, we developed a novel CEC assay, which does not require the centrifugal step.

## Methods

### Serum samples

Serum samples were obtained from healthy volunteers who provided written informed consent at Tokyo Medical and Dental University. The present study was approved by the Institutional Research Ethics Committee of the Faculty of Medicine of Tokyo Medical and Dental University (approval no. M2015-546).

### Measurement of serum lipids

The concentrations of total cholesterol (TC), triglycerides (TG), HDL-C, and low-density lipoprotein (LDL)-cholesterol (LDL-C) were measured using enzymatic kits (Minaris Medical Co., Ltd., Tokyo, Japan). These measurements were performed using a LABOSPECT 008 automatic analyzer (Hitachi High Technologies Corporation, Tokyo, Japan). TC levels were quantified using a commercial enzymatic assay kit, T-CHO (S) (Denka Co., Ltd., Tokyo, Japan).

### Preparation of HDL and apolipoprotein B-100-depleted serum

The HDL (1.063 < d < 1.210 g/ml) fraction was isolated from serum samples by ultracentrifugation as previously described [17]. The obtained HDL was dialyzed against PBS. The B-100-depleted serum (BDS) was prepared as described in our previous study [18]. Briefly, 40 µl of 20% polyethylene glycol 6000 in 200 mM glycine buffer (pH 7.4) was added to 100 µl of serum. After mixing and incubating at room temperature for 20 min, the supernatant was collected as BDS by centrifugation at 10000 rpm for 30 min.

### Preparation of ILGs and immobilized liposome-bound magnetic beads

ILGs were prepared as previously described with minor modifications [19]. Egg lecithin (10.6 mg) and cholesterol (2.3 mg) were dissolved in 6 ml chloroform, and 30 µl of 0.5 mM 4,4-difluoro-4-bora-3a, 4a-s-indacene-labeled cholesterol (BODIPY-labeled cholesterol; Avanti Polar Lipids Inc., Alabaster, AL, U.S.A.) was added to the solution. The lipid membrane formed under N_2_ gas was dissolved in diethyl ether and the solvent was evaporated. After performing this step twice, the lipid membrane was completely dried under N_2_ gas and suspended in 7 ml of 10 mM Tris-HCl (pH 7.4), containing 150 mM NaCl and 1 mM EDTA-2Na (buffer A). Then, 0.35 g of Dried Sephacryl S-300 gel beads (1 g; GE-Healthcare Bio-Sciences KK, Tokyo, Japan) was added to the liposome solution and allowed to react at room temperature for 30 min. The mixture was repeatedly frozen (–80°C) and thawed (room temperature, in water) for seven cycles to induce immobilization of the fluorescence-labeled liposomes in the gel beads (ILGs). Similarly, 2.45 g of magnetic beads (Core–shell ferrite powders, Powdertech Co., Ltd., Chiba, Japan) was added to the liposome solution and incubated at room temperature for 30 min. To immobilize the labeled liposomes, repeated freeze-thaw cycles were performed at the indicated times to produce immobilized liposome-bound magnetic beads (ILMs). Finally, both bead types were washed five times with buffer A and resuspended in 5 ml buffer A. The immobilized amount was evaluated by measuring the fluorescence of the washing buffer. The solutions were then stored in the dark at 4°C.

### CEC assay using ILGs and ILMs

The CEC assay was performed as previously described with minor modifications [19]. After uniformly suspending the stored ILGs or ILMs, 100 µl of each suspension was dispensed into a 2-ml tube. Then, 150 µl of various types of cholesterol acceptor solutions (final concentrations: 0.6–9.0 mg cholesterol/dl HDL or 0.2–5.0% BDS in percentage of serum) or buffer A (as a control) was added to the ILGs or ILMs. The mixture was incubated in the dark at room temperature (for ILG) or at 30°C (unless otherwise stated for ILM) for 16 h, and then the supernatants were separated from each bead using a spin-down centrifuge (for ILG) or a magnet (for ILM). The fluorescence intensity of the supernatant was measured at an excitation wavelength of 485 nm and an emission wavelength of 538 nm. These measurements were performed using a Fluoroskan Ascent (Thermo Fisher, Tokyo, Japan). The values were normalized using a reference BDS for every measurement, and the ratio was represented as the CEC.

### Statistical analysis

Correlation analyses were conducted using the Pearson’s test. The effects of stability and temperature were expressed as the mean ± SD and the mean + SD, respectively, and both values were tested using one-way ANOVA with Bonferroni correction. Differences were considered statistically significant at *P*<0.05.

## Results

### Liposome immobilized in porous magnetic beads

**Figure 1:**
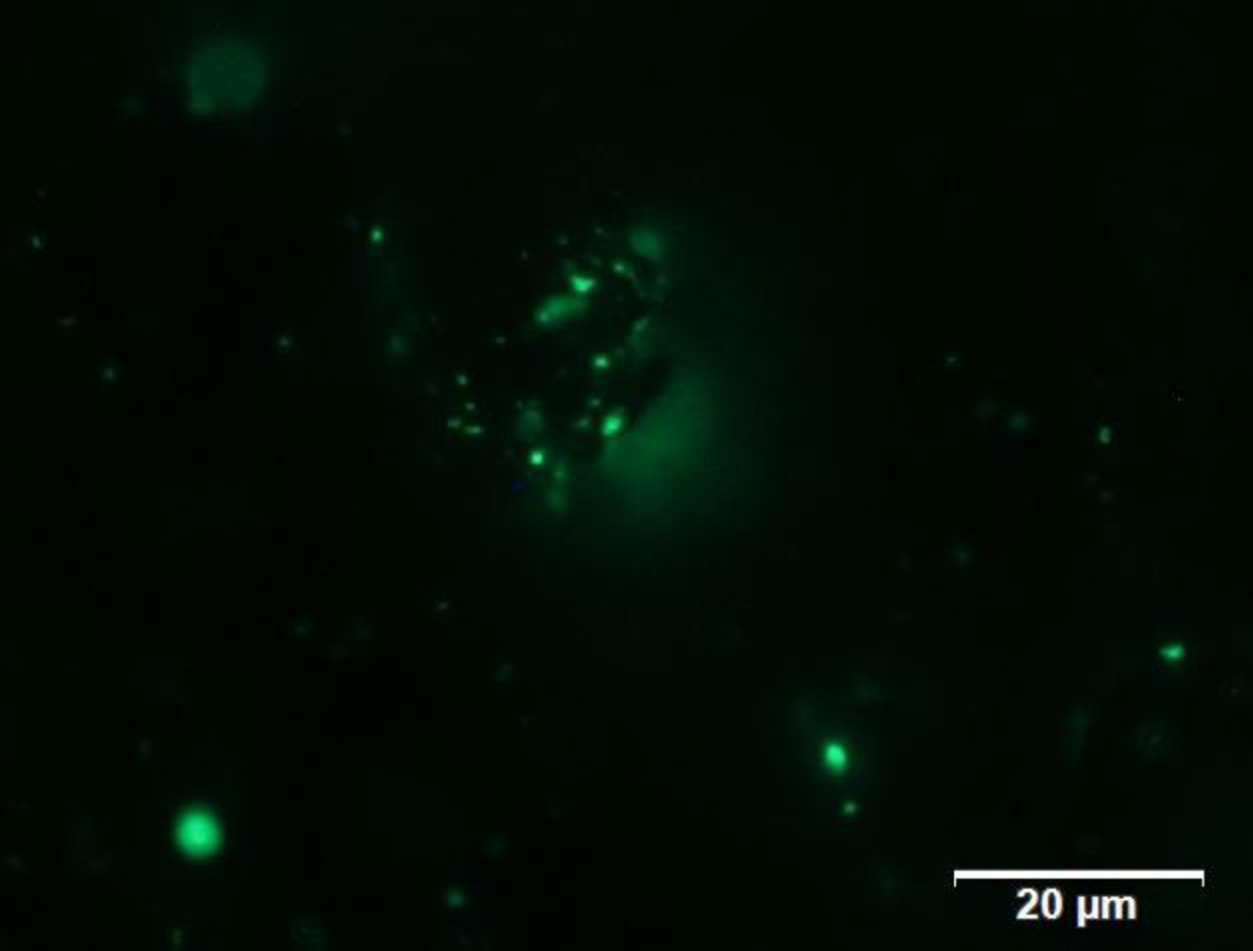
Liposome immobilized in the porous magnetic beads The magnetic beads treated with fluorescent cholesterol-labeled liposomes were observed using a fluorescence microscope (excitation filter; 460–480 GFP, emission filter; 495–540 GFP).

First, we confirmed whether fluorescent cholesterol-labeled liposomes were immobilized into porous magnetic beads. After immobilization treatment and washing, the treated magnetic beads were analyzed using a fluorescence microscope (Figure 1). Green fluorescence was observed as dots on the beads, indicating that the liposomes containing BODIPY-labeled cholesterol entered the beads and then fixed successfully.

### Evaluation of the amount of immobilized liposomes

**Figure 2:**
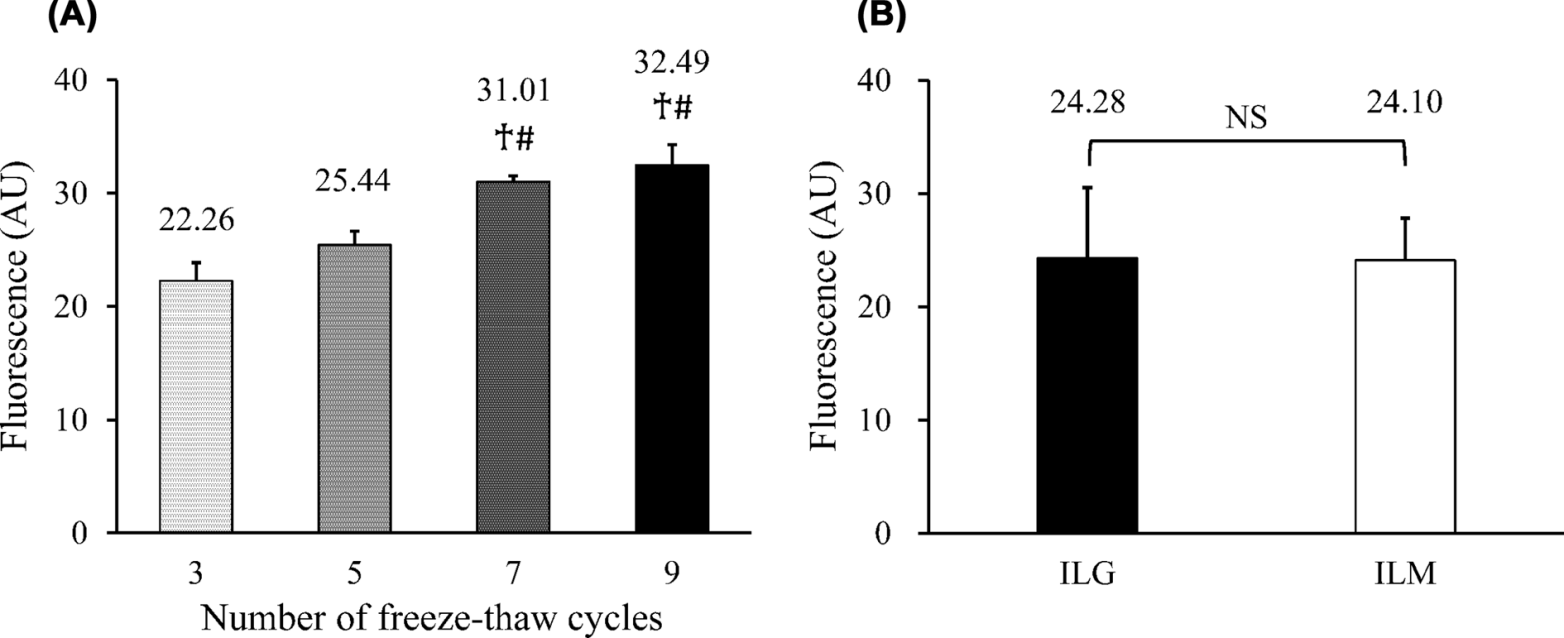
Evaluation of the amount of immobilized liposomes A liposome solution containing BODIPY-labeled cholesterol was mixed with the magnetic beads and treated with various numbers of freeze-thaw cycles. Finally, the frozen beads were thawed simultaneously and the fluorescence intensity of each supernatant was measured. (**A**) Difference in the intensity of the supernatant before and after freeze-thaw cycles (three, five, seven, and nine times). Three different mixtures were prepared in each cycle. One-way ANOVA with Bonferroni correction was used to compare the immobilized amounts. †*P*<0.05 vs three times, #*P*<0.05 vs five times. (**B**) Difference in the intensity of the supernatant before and after freeze-thaw cycles (seven times for ILG, *n*=6, and five times for ILM, *n*=10). A two-sample *t*-test was used for comparison between the ILG and ILM methods. Values are presented as mean + SD. Abbreviations: AU, arbitrary unit; NS, not significant.

Next, we estimated the amount of liposomes immobilized on the magnetic beads. The supernatants before and after several freeze-thaw cycles were collected, and the fluorescence intensity of each supernatant was measured. The difference in the intensity before and after freeze-thaw cycles was defined as the amount of immobilized liposomes on the beads. The amount of immobilized liposomes increased with repeated freeze-thaw cycles (Figure 2A). Moreover, we compared the amount of liposomes immobilized on the magnetic beads with those on the gel beads (for the ILG assay). The amount of immobilized liposomes on the magnetic beads after five freeze-thaw cycles was almost the same as that on the gel beads after seven cycles (Figure 2B). Therefore, we decided on five freeze-thaw cycles for preparing the ILM for the following experiments.

### Stability of the ILM

**Figure 3:**
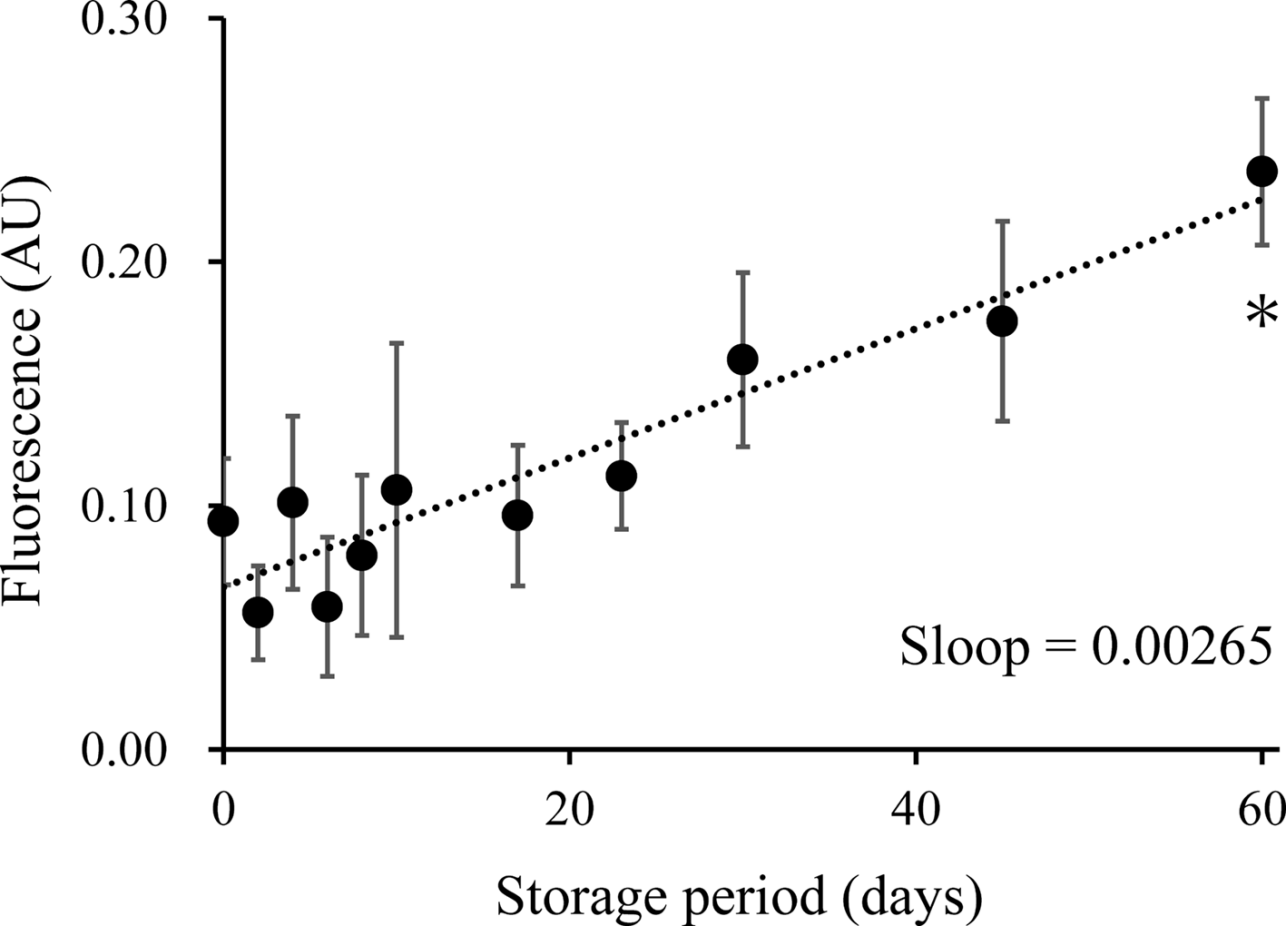
Stability of the ILM Similar to the efflux assay procedure, 100 µl of ILM suspension was mixed with 150 µl of Buffer A in multiple tubes and kept in the dark at 4°C. At various time points (0, 2, 4, 6, 8, 10, 17, 23, 30, 45, and 60 days), 100 µl of supernatant from the three tubes was transferred into new tubes and kept in the dark at 4°C. After 60 days of storage, the fluorescence intensity of the supernatant was measured in triplicate. Values are presented as mean ± SD. One-way ANOVA with Bonferroni correction was used for the comparison between the supernatant of day 0 and each measurement day. *P<0.05 vs day 0. Abbreviation: AU, arbitrary unit.

To estimate the stability of ILM, the ILM solutions were divided into tubes followed by mixing with buffer A as the efflux assay procedure, and then each tube was kept in the dark at 4°C for the indicated days. The fluorescence intensity of the supernatant increased from 0.093 arbitrary units on day 0 to 0.160 on day 30 and then increased to 0.237 after 60 days of storage (Figure 3). Only the supernatant on day 60 was significantly higher than that on day 0 (*P*<0.05).

### Comparison of fluorescence intensities and CEC values by the ILM method performed at between different incubation temperatures

**Figure 4:**
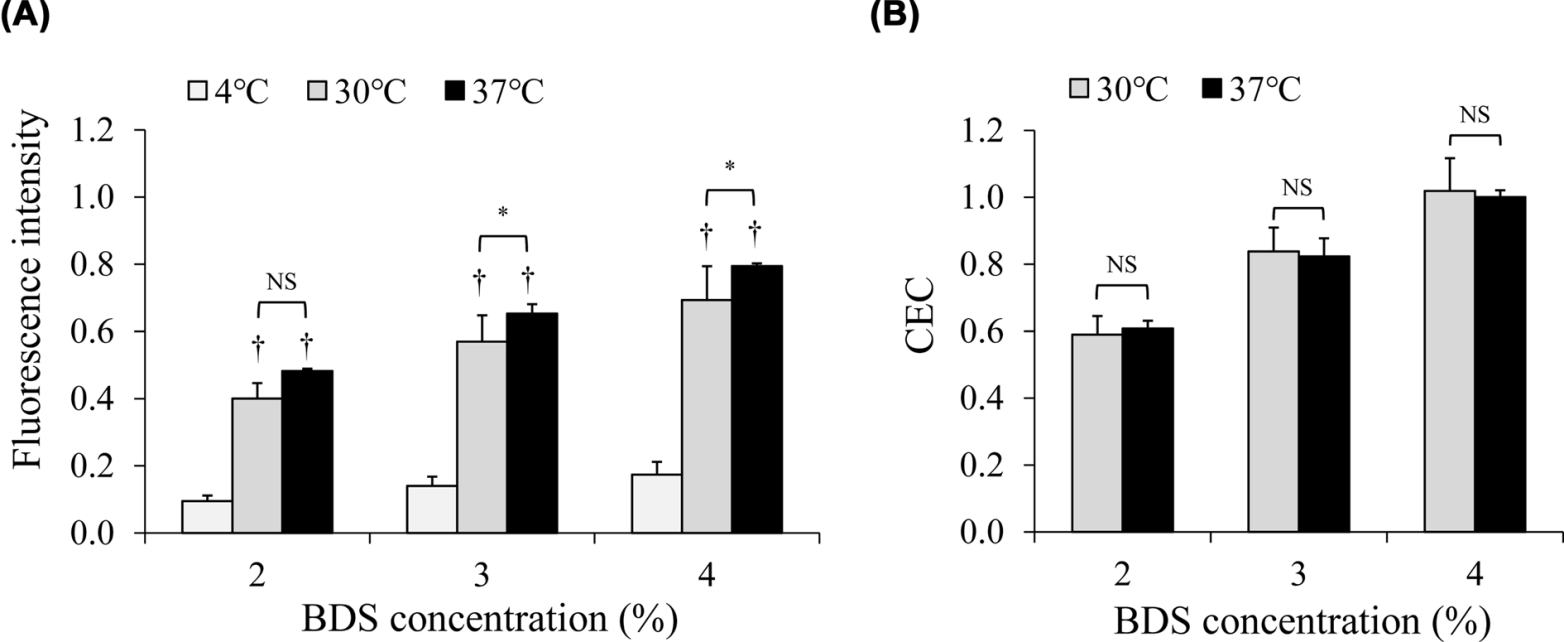
Comparison of fluorescence intensities and CEC values by the ILM method performed at between different incubation temperatures Various incubation temperatures (4, 30, and 37°C) were performed by the ILM method to evaluate CECs of BDSs (2%, 3%, and 4%). Differences in the fluorescence intensity of the supernatant (**A**) and calculated CECs (**B**) after incubation at between each temperature measurement. Values are presented as mean + SD. One-way ANOVA with Bonferroni correction was used for the comparison between the incubation temperatures. †*P*<0.05 vs at 4°C, *P<0.05. Abbreviation: NS, not significant.

To investigate differences in incubation temperatures, various levels of BDS (2%, 3%, and 4%) were tested by the ILM method performed at 4, 30, and 37°C. There were no large differences in fluorescence intensities obtained by ILM assay performed at between 30 and 37°C incubations regardless of BDS levels, while those at 4°C were quite low as shown in our previous report [15] (Figure 4A). Similarly, we further compared CEC values by the ILM method performed at between 30 and 37°C. Consequently, there was no significant difference in CEC values (Figure 4B). In order to confirm the comparability of the ILM method with the ILG method, the following experiments by the ILM method were performed at 30°C that was close to the condition of ILG method (room temperature).

### Repeatability

Using the ILMs, within-day and between-day analyses were performed to evaluate the ILM method repeatability. When three serum samples were run 20 times each, the coefficients of variation were less than 10.0% (Table 1).

**Table 1:** Repeatability of CEC assay

Within-day			Between-day		
	CEC (mean ± SD)	CV (%)		CEC (mean ± SD)	CV (%)
Sample 1	0.756 ± 0.044	5.8	Sample 4	0.806 ± 0.063	7.7
Sample 2	0.839 ± 0.051	6.1	Sample 5	0.873 ± 0.080	9.2
Sample 3	0.952 ± 0.063	6.6	Sample 6	0.872 ± 0.080	9.2

We verified the within-day and between-day repeatability. For verification, 20 tubes were tested using three serum samples. Values are reported as mean ± SD (*n*=20).

### Effect of washing ILM before CEC assay

**Figure 5:**
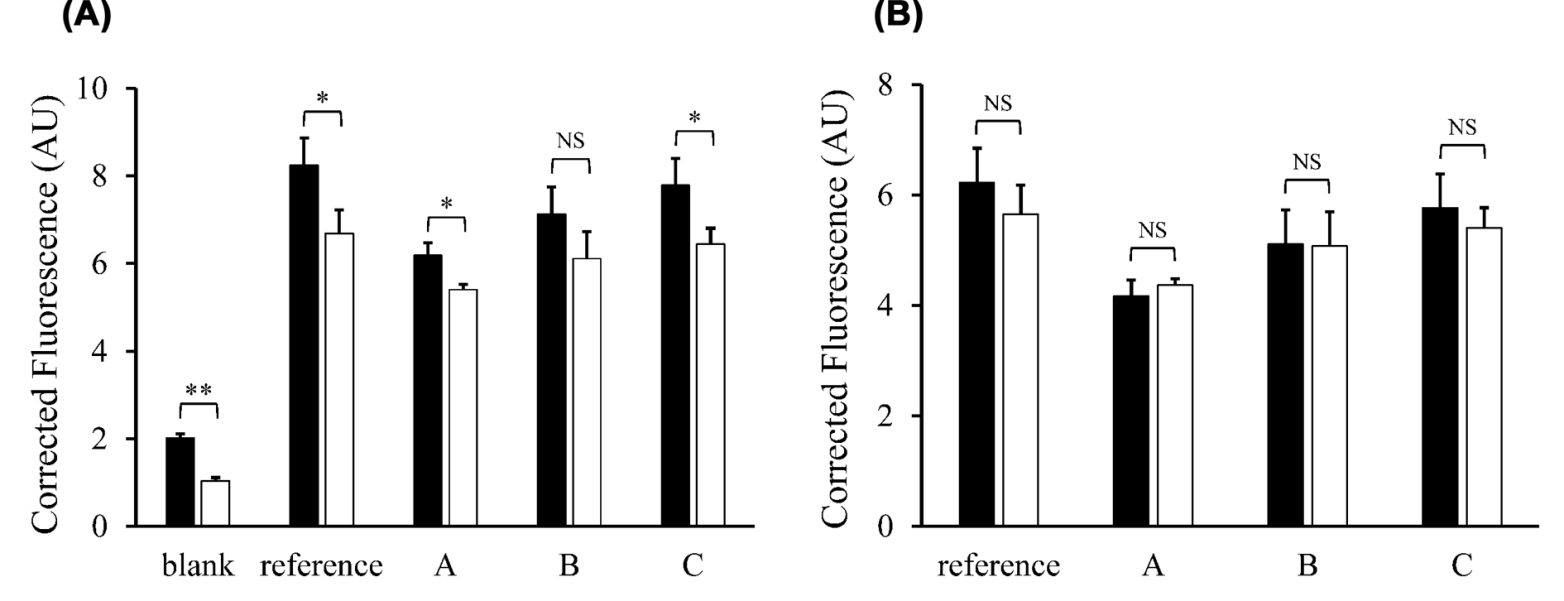
Effect of washing ILM before CEC assay The CECs of buffer A (blank), the reference serum, and three serum samples (A, B, and C) were determined using the same ILM lot with (white bar) and without (black bar) washing immediately before the assays (**A**). The fluorescence was corrected by subtracting the blank value (basic diffusion) from each experimental value. (**B**) Values are presented as mean + SD (*n*=3). Student’s *t*-test (unpaired, two-tailed) was used to compare the fluorescence intensity with and without washing; *P<0.05, **P<0.01. Abbreviations: AU, arbitrary unit; NS, not significant.

Before CEC measurement, we verified whether the stocked ILMs required a washing process to remove the diffused fluorescence-labeled cholesterol from the magnetic beads. To prepare the washed ILMs, after removing the supernatant from the ILM solution stored in the dark at 4°C, the ILMs were washed twice with buffer A and resuspended in 5 ml buffer A. The effects on CEC of BDSs from three healthy subjects were verified by comparing them with and without washing ILMs. As a result, the fluorescence intensity of the supernatant was significantly higher in the sample without washing ILMs (Figure 5A). However, when the fluorescence intensities from those BDSs were subtracted from those from buffer A (background fluorescence), the differences between with and without washing ILMs were offset (Figure 5B). The following CEC assay using HDL and BDS was performed with background fluorescence subtraction.

### Linearity of the ILM method and correlation with the ILG method

**Figure 6:**
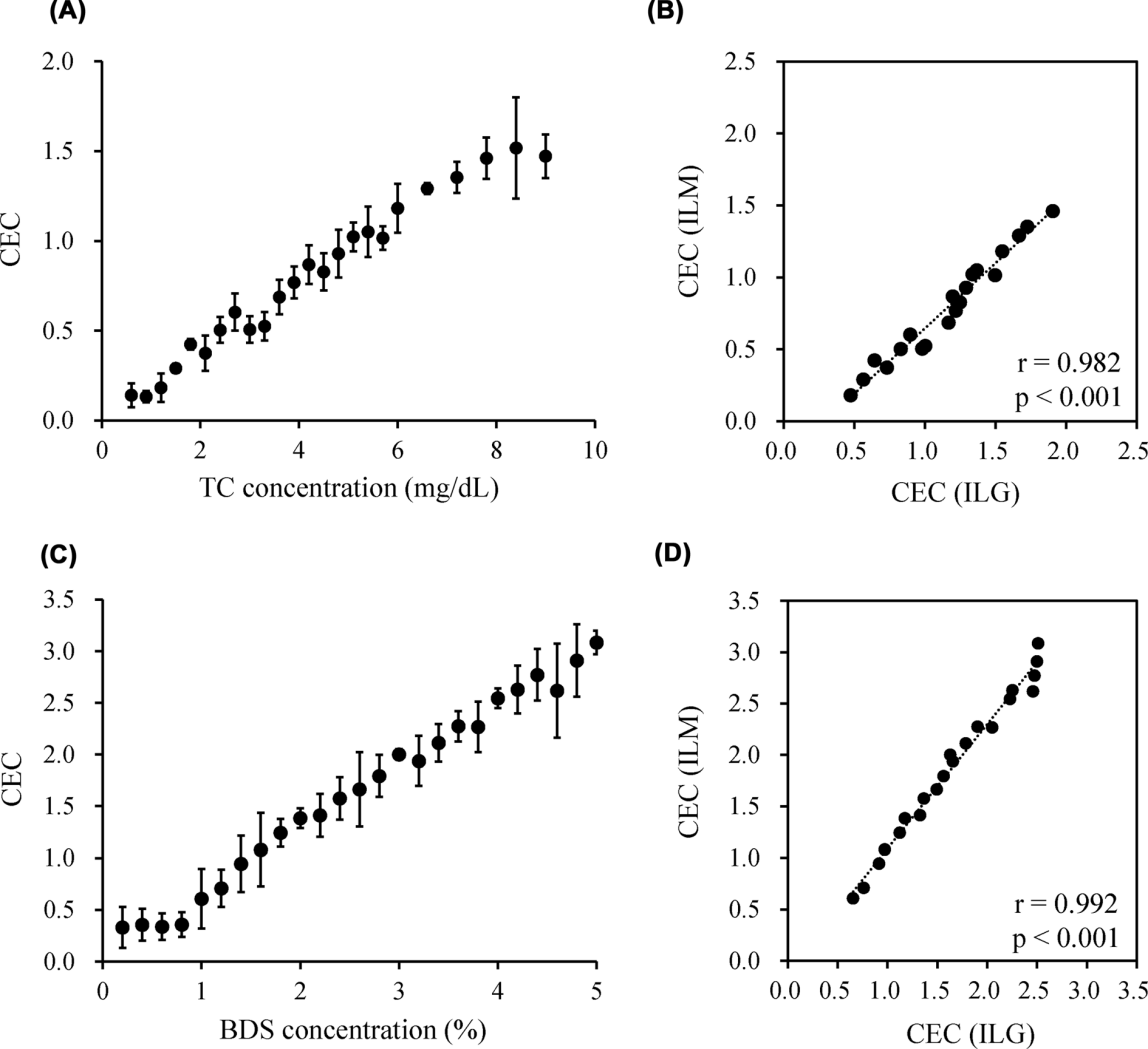
Basic performance of the ILM method To confirm a concentration dependency of CEC by the ILM method, CEC of 24- and 25-dilution series of HDL (0.6–9.0 mg cholesterol/dl) (**A**) and BDS (0.2–5.0% in percentage of serum) (**C**), respectively, were evaluated. Using those values within the range of linearity from the above results (HDL; *n*=20, BDS; *n*=21), the correlation with the ILG method was confirmed (**B,D**). Values are presented as mean ± SD. The Pearson’s test was used for comparisons.

To evaluate the basic performance of the ILM method, CECs of various levels of HDL (0.6–9.0 mg cholesterol/dl) and BDS (0.2–5.0% BDS in percentage of serum) samples prepared by sequential dilution were determined by the ILM method. As a result, CECs of HDL and BDS increased in a concentration-dependent manner in the range of 1.2–7.8 mg cholesterol/dl and 1.0–5.0%, respectively (Figures 6A and C). In contrast, no concentration-dependency was observed in the range of 0.6–1.2 mg cholesterol/dl HDL and 0.2–1.0% BDS, respectively.

Using samples whose CEC values were within the range of linearity, the correlations between this novel ILM method and the conventional ILG method were investigated. As a result, CEC values obtained by the ILM method were well correlated with the ILG method in both HDL and BDS samples (*r* = 0.982 and *r* = 0.992, respectively) (Figures 6B and D).

### Correlations of BDS-CECs from healthy subjects between the ILM and ILG methods

**Figure 7:**
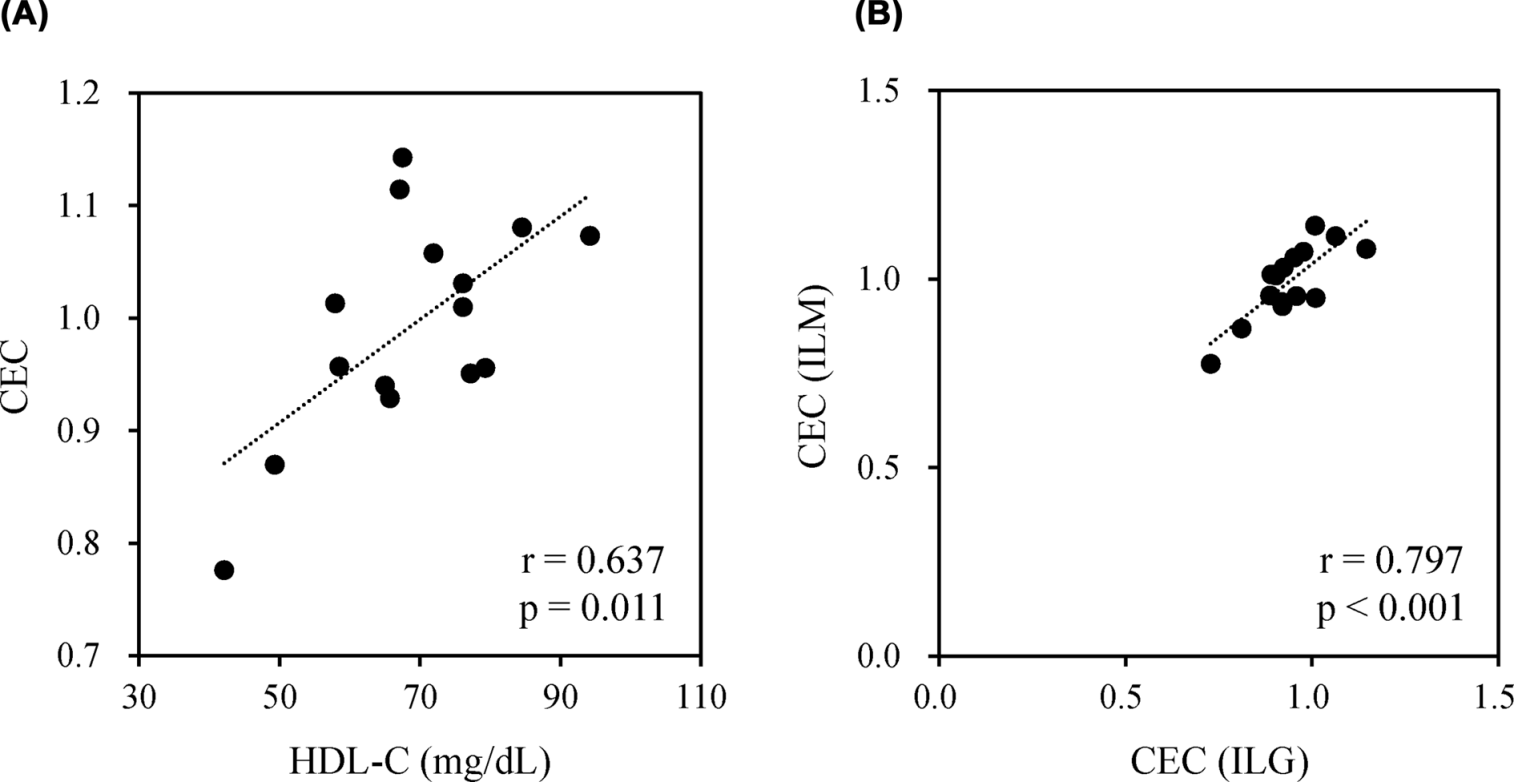
Correlations of BDS-CECs from healthy subjects The CECs of 15 healthy human serum samples with various HDL-C concentrations were measured using both the ILG and ILM methods. The CECs of the ILM method were measured at a final BDS concentration of 3.5%, and those of the ILG method were measured at a final BDS concentration of 2.0%. The correlation of CEC by ILM with HDL-C concentration (**A**) and with CEC by the ILG method (**B**) was examined. The Pearson’s test was used for comparison.

In order to investigate the relationship between CEC values and HDL-C, BDS-CECs from 15 healthy subjects with various serum HDL-C concentrations (42–94 mg/dl) (Table 2) were determined using both the present method (ILM) and the conventional method using gel beads (ILG). First, we attempted to perform CEC measurements by the ILM method using 2% BDSs in the same manner as the ILG method. However, as shown in Figure 6A, because low HDL-C (<1.2 mg/dl) was below the detection limit, precise measurement of serum samples from healthy subjects with low HDL-C concentration (<60 mg/dl) was not feasible when it was performed using 2% BDSs. Therefore, CEC was remeasured at a BDS concentration of 3.5% in the ILM method, which was supposed to be measurable up to a serum HDL-C concentration of 34 mg/dl. Consequently, CEC values by the ILM method (3.5% BDS) showed a relatively strong correlation with HDL-C (*r* = 0.637, Figure 7A) and CEC by the ILG method (*r* = 0.797, Figure 7B). Notably, there were large differences in CEC values among some BDS samples from subjects with similar HDL-C concentrations (approximately 65 mg/dl) (Figure 7A), which suggested that a similar amount of HDL showed a different antiatherosclerotic capacity.

**Table 2:** Lipid profiles of the subjects in the correlation assay (*n*=15)

	TG	TC	HDL-C	LDL-C
mean ± SD (mg/dl)	60.8 ± 25.4	174.5 ± 18.6	68.8 ± 13.5	93.5 ± 11.0
max (mg/dl)	114.4	213.2	94.2	114.0
mix (mg/dl)	31.6	131.1	42.1	76.9

Abbreviations: HDL-C, high-density lipoprotein cholesterol; LDL-C, low-density lipoprotein cholesterol; TC, total cholesterol; TG, triglyceride.

## Discussion

CVD is the leading cause of death all over the world, making early detection and diagnosis of this asymptomatic disease important for survival. CEC has attracted a great deal of attention as a biomarker for CVD risk, in addition to HDL-C. Determination of CEC has already been reported to be a better biomarker than HDL-C [12,13] and is expected to be useful for clinical CVD risk assessment. However, the conventional CEC measurement assay requires cultured cells and radioisotope-labeled cholesterol [14], which are not practical in clinical settings. To solve this problem, we developed a new CEC assay in which gel beads and BDS were used instead of cells and radioisotopes, respectively [15]. However, this method requires the isolation of a supernatant-containing fluorescence-labeled cholesterol from gel beads using a spin-down centrifuge. This centrifugal step requires great care not to inhale the gel, as gel beads easily swirl up with slight vibrations. Therefore, this assay was not suitable for the installation of an automated analyzer. In the present study, we developed a method that does not require centrifugation steps during the assay. We focused our attention on magnetic beads because they have been widely used in automatic immunoassay analyzers for the separation of free and antibody-bound components. Using magnetic beads, it would be easier to separate the supernatant from the CEC assay and it would be possible to mount it on an automated analyzer. In the present study, we developed and verified a new method for measuring CEC using magnetic beads.

First, it is necessary to successfully immobilize liposomes, including fluorescence-labeled cholesterol, onto the magnetic beads. For this purpose, we selected unique porous magnetic beads whose holes could be used to confine the liposomes as gel beads do. As expected, freeze-thaw cycles enlarged the liposomes inside the magnetic beads rather than on the surface, as observed by fluorescence microscopy, and the reduction in fluorescence intensity of the supernatant was also confirmed. Regarding the immobilization rate, the amount of immobilized liposomes was approximately the same when the number of freeze-thaw cycles in the ILG method was seven, and the number in the ILM method was five. Based on this result, we estimated that the ILM method has a higher liposome immobilization efficiency than the ILG method. The dissociation of immobilization efficiency was attributed to the difference in hole size between the gel beads and the magnetic beads, which created a gap in quantity.

In addition, when measuring CEC using the ILM method in a clinical setting, the ILM solution should be stable for at least a few months as a manufactured product. Therefore, the stability of the prepared ILM was confirmed. The ILMs were stable for 45 days, and it was confirmed that the fluorescence of the supernatant tended to increase slightly after 45 days. Although this phenomenon was also confirmed using the ILG method [10], the increased fluorescence intensity due to ILGs was lower than ILMs. This is because small-sized liposomes or free cholesterol that are also immobilized on ILM can be easily detached from the beads. However, even if diffusion occurs, the degree was quite small compared with the amount of the immobilized BODIPY-cholesterol into the magnetic beads (Figure 2A), and our current results suggest that blank subtraction or ILM washing is effective.

To investigate the basic performance of CEC measurement by ILM, we measured the CEC of HDL or BDS samples diluted in 24 or 25 series, respectively. It was confirmed that CEC increased in a concentration-dependent manner; however, in both HDL and BDS, linearity could not be confirmed in the highly diluted region. This is because the amount of cholesterol extracted by HDL was too low to measure the CEC in the diluted samples. In addition, in the correlation diagram of BDS (Figure 6D), parallel samples were confirmed at high CEC concentrations, which was attributed to the existence of the upper limit of CEC measured by the ILG method at high BDS concentrations. However, the linearity range of HDL-C (1.2–7.8 mg/dl) shown in the present study reflects 34–223 mg/dl as serum HDL-C when the ILM method is performed using 3.5% BDS. Therefore, our method has sufficient performance to evaluate the CEC of HDL-C concentration expected in clinical patients.

Moreover, CEC values obtained using the ILM method showed good correlation with those obtained using the ILG method. However, in the HDL samples, the CEC of the ILM method was lower than that of the ILG method. We suggest that the efflux of HDL and BDS might be different in the ILM, and since CEC is expressed as the ratio of reference serum, it is possible that divergence has occurred. BDS differs from HDL in that it contains many serum proteins in addition to HDL. In the ILG method, it is known that globulin has the ability to efflux cholesterol [15]. In addition, previous reports have described the effect of serum proteins, especially albumin, on CEC obtained using BDS as the cholesterol acceptor [16,20,21]. Hence, these serum proteins might have extracted cholesterol from the ILM, and this deviation may have occurred. On the other hand, in the correlation of BDS, since BDS was used as a reference serum, less scattered data were observed in the correlation diagram. As a countermeasure against this, when measuring HDL using the ILM method, we suggest that using a reference HDL may eliminate the dissociation of the correlation with the ILG method, which we will implement in the future. From these results, it became clear that CEC of HDL and BDS can be measured using ILM, and we evaluated the CEC of the serum of 15 healthy subjects with various HDL-C concentrations. Similar to the ILG method, when CEC measurement was performed at a BDS concentration of 2% in the ILM method, a deviation from the ILG method was observed in the sample with a low HDL-C concentration, suggesting that CEC may not be accurately reflected. Furthermore, from the results of the dilution series of HDL (Figure 6A), it was predicted that accurate measurement would not be possible for samples with a serum HDL-C concentration of less than 60 mg/dl. Therefore, we decided to measure the CEC of healthy subjects at a BDS concentration of 3.5%, which can measure serum HDL-C concentrations of up to 34 mg/dl. The results showed that HDL-C concentrations correlated with CEC and strongly correlated with the ILG method. The correlation of HDL-C was *r* = 0.619 in the previously reported the ILG method [15], whereas our method showed almost the same result (*r* = 0.637).

Furthermore, there were samples with the same HDL-C level but different CEC values. In other words, even if the amount of HDL is the same, the function of each is different, and it can be said that measuring CEC may be more useful in predicting the risk of atherosclerosis. Previous study using the conventional CEC method reported that CVD patients with very high HDL-C concentrations showed significantly lower CEC [22]. Using the ILM method allowing high-throughput measurement with enough performance for CEC evaluation with a wide range of HDL-C levels, in the future, we plan to verify whether CEC evaluation is possible for healthy subjects and patient serum with various HDL-C concentrations.

The limitation of the present study is that the ILM method (also the ILG method) uses noncellular sources as cholesterol donor, and there will be a change or fluctuation in different batches. However, use of reference BDS is effective to correct the CEC values obtained from each separate experiment as shown in our previous study [15]. In addition, in other words, calibration correction factor to determine the relative fluorescence unit for this assay is available easier than those for fluorescence-labeled cholesterol-loaded cells. As for the relationship between beads-based methods (the ILG and ILM methods) and the conventional cell-based method, the ILM method does not involve transporter-mediated efflux, which is reflected in cellular methods. However, it has already been reported that the CEC of the conventional cellular method is well correlated with the ILG method, and CEC values of BDS reflect the HDL-CEC [15,16]. Moreover, when CEC values obtained by the ILG and conventional methods were compared between HDL_2_ and HDL_3_, similar trends (HDL_2_ > HDL_3_) in both methods were observed, which suggested that the specificities of the ILG method and cell-based method to HDL were similar, whereas the ILG method showed different behavior in CEC values of apolipoprotein E-containing HDL and free apoA-I from the cell-based method: CEC values by the ILG method were quite lower than those by the cell-based method [16]. Since the ILM and ILG methods have a same specificity to HDL as mentioned above, CEC by the ILM method is also thought to generally reflect that of the conventional method using the major HDL subclasses such as HDL_2_ and HDL_3_, but the ILM method has some limitations such that it does not reflect CEC of lipid-free apoA-I. We believe that the ILM method has still advantages enough in terms of the simplicity and suitability for clinical use. However, it has not yet been confirmed whether CEC values obtained by ILM reflect the efflux capacity via ABCA1 and ABCG1 transporters *in vivo*. Further investigation of ILM CEC by basic and clinical research using various patients is required in the future.

## Conclusion

The present study demonstrates that we succeeded in immobilizing liposomes containing fluorescence-labeled cholesterol on magnetic beads. In addition, we confirmed a concentration-dependent increase in HDL and BDS samples, and suggest that this method can be used to evaluate the CEC of patients. In the future, it will be necessary to verify whether CEC measured using the ILM method can be evaluated in various types of patients. We also aim for the clinical application of CEC measurement using the ILM method.

## Data Availability

The data used to support the findings of the present study are available from the corresponding author (Ryunosuke Ohkawa, Graduate School of Medical and Dental Sciences, Tokyo Medical and Dental University (TMDU), ohkawa.alc@tmd.ac.jp) upon request.

## Abbreviations

ABC: ATP-binding cassette transporter
ANOVA: analysis of variance
apoA-I: apolipoprotein A-I
AU: arbitrary unit
BDS: B-100 depleted serum
CEC: Cholesterol efflux capacity
CVD: cardiovascular disease
HDL: high-density lipoprotein
HDL-C: HDL cholesterol
ILG: immobilized liposome-bound gel bead
ILM: immobilized liposome-bound magnetic bead
LDL: low-density lipoprotein
LDL-C: LDL-cholesterol
NS: not significant
SD: standard deviation
SR-B1: scavenger receptor class B type 1
TC: total cholesterol
TG: triglyceride
